# Auricular Acupuncture at the “Shenmen” and “Point Zero” Points Induced Parasympathetic Activation

**DOI:** 10.1155/2013/945063

**Published:** 2013-06-04

**Authors:** Young-Chang P. Arai, Yoshikazu Sakakima, Jun Kawanishi, Makoto Nishihara, Akihiro Ito, Yusuke Tawada, Yuki Maruyama

**Affiliations:** ^1^Department of Surgery, Toki General Hospital, Gifu 509-5193, Japan; ^2^Multidisciplinary Pain Centre, Aichi Medical University, School of Medicine, 21 Karimata, Aichi-gun, Aichi, Nagakute 480-1195, Japan

## Abstract

*Purpose*. Since auricular acupuncture is a diagnostic and treatment system based on normalizing the body's dysfunction, auricular acupuncture has been applied for pain relief, relaxation, and so on. These techniques would modulate the autonomic nerve system, thereby inducing the above-mentioned effects. The aim was to see the effect of auricular acupuncture applied to the “Shenmen” and “Point Zero” points on the postoperative heart rate variability (HRV). *Methods*. Twenty-six patients who underwent hemicolectomy under general anesthesia were randomized into the control or the acupuncture group. After the operation and before emergence, the acupuncture group received auricular acupuncture. An electrocardiographic unit was placed for recording the autonomic nervous activities. *Results*. The low frequency (LF)/high frequency (HF) ratio of HRV increased (*P* = 0.0007) in the control, but the ratio in the acupuncture did not change. There were significant differences between the ratios of the two groups at 3 : 00, 4 : 00, and 5 : 00. HF of the acupuncture group tended to be higher. HFs of the acupuncture group were significantly higher than those of the control group at 3 : 00, 4 : 00, and 5 : 00. *Conclusion*. Auricular acupuncture kept the LF/HF ratio at lower levels and HF at higher levels during postoperative period in the patients who had undergone hemicolectomy.

## 1. Introduction

Pain relief, relaxation, and the other effects are provided by acupuncture and acupressure techniques using the traditionally used acupuncture points [1–4]. Auricular acupuncture is a diagnostic and treatment system based on normalizing the body's dysfunction [5]. Also, auricular acupuncture has been applied in order to relieve postoperative pain and improve neurorehabilitation and insomnia [6–8]. Moreover, our previous case series showed that after agitated patients received auricular acupuncture at tranquilizing points, they did not show postoperative problematic behaviors such as agitation [9]. Some studies have suggested that these techniques would modulate the reticular formation and the autonomic nerve system, thereby inducing the above-mentioned effects [5].

 Frequency-domain analysis of heart rate variability (HRV) is a sophisticated noninvasive tool for the assessment of autonomic nervous system (ANS) regulation of the heart [1, 3, 10, 11]. Frequency fluctuations in the range of 0.04–0.15 Hz (low frequency, LF) are considered to be markers of sympathetic nerve activity, and high frequency (HF) fluctuations in the range of 0.15–0.4 Hz are considered markers of parasympathetic nerve activity [1, 3, 10]. The LF/HF ratio is considered to show sympathovagal balance or reflect the sympathetic modulation [1, 3, 12]. Under various conditions such as physical and mental stress, the activity of the ANS changes and these parameters change.

 We hypothesized that auricular acupuncture at the “Shenmen” and “Point Zero” points [9, 13] would tranquilize the mind of patients during postoperative period, thereby changing autonomic nervous activity. In the present study, we therefore investigated the effect of auricular acupuncture applied to the “Shenmen” and “Point Zero” points on the postoperative HRV in patients who underwent hemicolectomy for an ascending or descending colon cancer.

## 2. Methods

After obtaining approval from the Ethics Committees of our institution and written informed patient's consent, 26 American Society of Anesthesiologists physical status I or II patients presenting for hemicolectomy under general anesthesia combined with epidural anesthesia were enrolled in the present study from February 2010 to November 2010. Patients who had a history of central nervous system or cardiovascular system dysfunction were not invited to participate in the present study.

**Figure 1:**
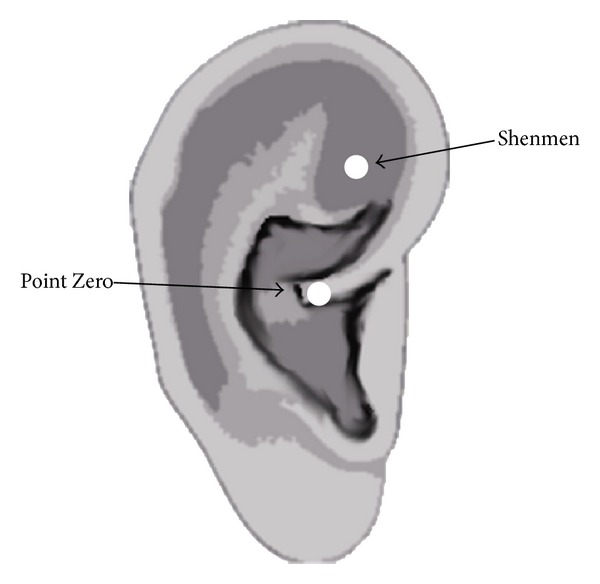
The locations of the “Shenmen” and “Point Zero” points.

 Patients were randomized into two groups, using sealed envelopes: the control group received no treatment; the acupuncture group postoperatively received occlusive press needles (Pyonex-small; Seirin, Japan) at the “Shenmen” and “Point Zero” points (Figure 1) [9, 13] at both auricles. On arrival in the operation room, all patients had standard monitoring in place (noninvasive arterial pressure, electrocardiogram (ECG), and pulse oximetry). With the patients in the right lateral position, a 20-gauge epidural catheter was placed after identification of the epidural space at the Th10-11 interspace using a 17-gauge Tuohy needle. Then, general anesthesia was induced with fentanyl 100 *μ*g and propofol 160 mg. Anesthesia was maintained using 1.5–2% sevoflurane and epidural 2% lidocaine 8 mL. They received fentanyl 20 *μ*g h^−1^ and 1% lidocaine 2 mL h^−1^ epidurally for the management of postoperative pain. After the operation and before emergence, patients of the acupuncture group received occlusive press needles. All patients were not told whether or not they had received auricular acupuncture. A palm-sized electrocardiographic unit (Active Tracer AC300, GMS, Tokyo, Japan) was placed on all patients for continuous recording of the variation of autonomic nervous activities [14, 15]. The unit was worn in a pouch at the waist with three electrodes taped to the chest until the next day of the operation.

Data recorded in the palm-sized electrocardiographic unit was analyzed for heart rate variability (HRV) by the maximum entropy method (CHIRAM, Suwa Trust Japan, Japan) [14, 15]. The R-R intervals (RRI) were obtained every 5 minutes. The two components of power of the RRI (ms.ms); LF (0.04–0.15 Hz) and HF (0.15–0.5 Hz), were calculated. Heart rate (HR), the LF and the HF values, and the LF/HF ratio of HRV were analyzed.

Data are presented as the median (range), number or the median with the 25th and 75th percentiles. The demographic data was analyzed by the Mann-Whitney *U* test or chi-square test. The Friedman test followed by Dunn's method for multiple comparisons was used for intragroup comparison because of the nonnormal distribution. The Mann-Whitney *U* test was used to analyze intergroup data. *P* < 0.05 was considered statistically significant.

## 3. Results

Twenty-six patients were assigned to the control, or acupuncture group. The two groups were comparable in terms of patients' characteristics (Table 1). All patients did not show postoperative problematic behavior.

**Figure 2:**
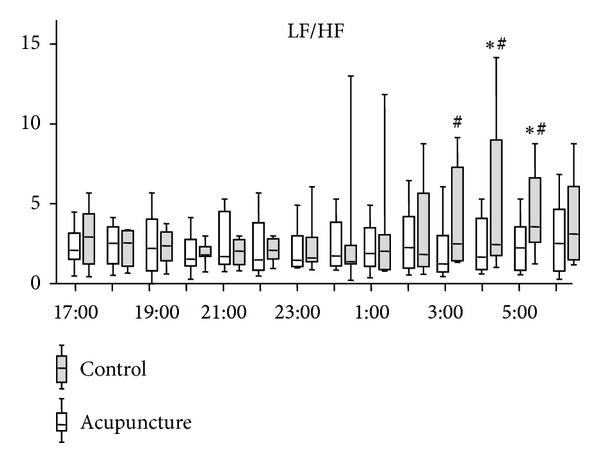
Time course of change in low frequency (LF)/high frequency (HF) ratio of heart rate variability. Horizontal bars represent medians, boxes represent the 25th and 75th percentile ranges, and T bars represent the 5th and 95th percentile ranges. *Different from 23 : 00 (*P* < 0.05). ^#^Different from the acupuncture group (*P* < 0.05).

**Figure 3:**
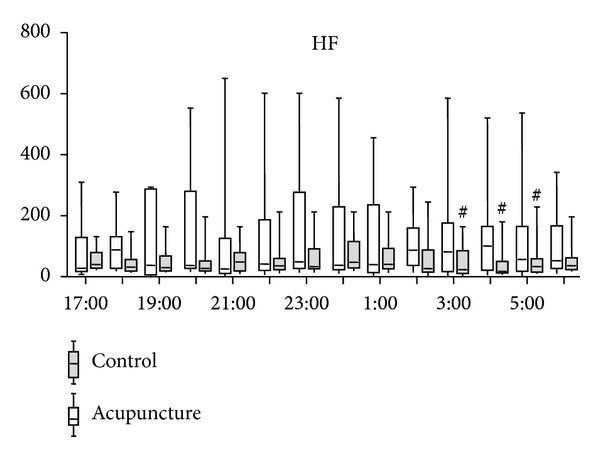
Time course of change in high frequency (HF) of heart rate variability. Horizontal bars represent medians, boxes represent the 25th and 75th percentile ranges, and T bars represent the 5th and 95th percentile ranges. ^#^Different from the acupuncture group (*P* < 0.05).

 In the control group, the LF/HF ratio of HRV started to increase around 1 : 00 (*P* = 0.0007) and the ratio significantly increased at 4 : 00 and 5 : 00 compared to that at 23 : 00 (Figure 2). In contrast, the LF/HF ratio in the acupuncture group did not change (*P* = 0.8489) (Figure 2). There were significant differences between the ratios of the two groups at 3 : 00, 4 : 00, and 5 : 00. HF of the acupuncture group tended to be higher than that of the control group (Figure 3). HFs of the acupuncture group were significantly higher than those of the control group at 3 : 00, 4 : 00, and 5 : 00 (Figure 3).

## 4. Discussion

Since acupuncture is a diagnostic and treatment system based on normalizing the body's dysfunction, acupuncture has been applied in order to provide pain relief, relaxation, and the other effects [1–8]. Experimental and clinical investigations have indicated that afferent input in somatic nerve fibers has a significant effect on the autonomic nervous system and hormones. Several studies have investigated the effect of acupuncture on the sympathetic nervous system. In contrast, only a few have explored its effect on the parasympathetic nervous system [16]. Some studies have suggested that these techniques would modulate the reticular formation and the autonomic nerve system [5], thereby inducing the above-mentioned effects. Although there has been a controversy on the effect of acupuncture on the parasympathetic nervous activity, several studies have shown that auricular acupuncture influences parasympathetic nerve activity [17, 18].

 The present study showed that auricular acupuncture kept the LF/HF ratio at lower levels and HF at higher levels during postoperative period in the patients who had undergone hemicolectomy. The data indicate that occlusive press needles at the “Shenmen” and “Point Zero” points at both auricles increased parasympathetic nerve activity, which is consistent with the above-mentioned studies.

 In some pathophysiologically disturbed cases, we observe neuropsychiatric symptoms such as epileptic seizure and delirium [19, 20]. Several studies have shown that epilepsy occurs in line with autonomic imbalance, an increased sympathetic activity and a reduced parasympathetic activation [21, 22]. Also, several reports have described that induction or withdrawal of some drugs induced simultaneously delirium and autonomic dysfunction, an increased sympathetic activity [23–25]. Thus, the procedures increasing parasympathetic tone might treat and alleviate these diseases. Our previous case series showed that after agitated patients received auricular acupuncture at tranquilizing points, they did not show postoperative problematic behavior such as agitation [9]. We therefore postulate that, as the present study demonstrated, auricular acupuncture at tranquilizing points had induced the activation of parasympathetic nerve tone, thereby preventing postoperative agitation in the case series.

 In conclusion, auricular acupuncture kept the LF/HF ratio at lower levels and HF at higher levels during postoperative period in the patients who had undergone hemicolectomy.

## Figures and Tables

**Table 1:** Demographic data of patients. Values are the median (range) or number.

	Control group	Acupuncture group	*P*
	(*n* = 13)	(*n* = 13)	
Age (year)	62.5 (37–72)	54 (40–77)	0.8775
Sex (M/F)	8/5	9/4	0.7534
Weight (kg)	50 (43–68)	53.5 (36–70)	0.5308
